# Motivation, students’ needs and learning outcomes: a hybrid game-based app for enhanced language learning

**DOI:** 10.1186/s40064-016-2971-1

**Published:** 2016-08-09

**Authors:** Anke Berns, José-Luis Isla-Montes, Manuel Palomo-Duarte, Juan-Manuel Dodero

**Affiliations:** 1 Departamento de Filología Francesa e Inglesa, Facultad de Filosofía y Letras, Universidad de Cádiz, Avda. Gómez Ulla s/n, 11002 Cádiz, Spain; 2 Departamento de Ingeniería Informática. Escuela de Ingeniería, Universidad de Cádiz, Avda. de la Universidad n° 10, 11519 Puerto Real, Cádiz, Spain

**Keywords:** Language learning, Learning needs, Hybrid game-based app, Digital native, Motivation, Sustainability, Smart mobile device, Collaborative learning, Learning

## Abstract

In the context of European Higher Education students face an increasing focus on independent, individual learning—at the expense of face-to-face interaction. Hence learners are, all too often, not provided with enough opportunities to negotiate in the target language. The current case study aims to address this reality by going beyond conventional approaches to provide students with a hybrid game-based app, combining individual and collaborative learning opportunities. The 4-week study was carried out with 104 German language students (A1.2 CEFR) who had previously been enrolled in a first-semester A1.1 level course at a Spanish university. The VocabTrainerA1 app—designed specifically for this study—harnesses the synergy of combining individual learning tasks and a collaborative murder mystery game in a hybrid level-based architecture. By doing so, the app provides learners with opportunities to apply their language skills to real-life-like communication. The purpose of the study was twofold: on one hand we aimed to measure learner motivation, perceived usefulness and added value of hybrid game-based apps; on the other, we sought to determine their impact on language learning. To this end, we conducted focus group interviews and an anonymous Technology Acceptance Model survey (TAM). In addition, students took a pre-test and a post-test. Scores from both tests were compared with the results obtained in first-semester conventional writing tasks, with a view to measure learning outcomes. The study provides qualitative and quantitative data supporting our initial hypotheses. Our findings suggest that hybrid game-based apps like VocabTrainerA1—which seamlessly combine individual and collaborative learning tasks—motivate learners, stimulate perceived usefulness and added value, and better meet the language learning needs of today’s digital natives. In terms of acceptance, outcomes and sustainability, the data indicate that hybrid game-based apps significantly improve proficiency, hence are indeed, effective tools for enhanced language learning.

## Background

Vocabulary input is a primary learning need, especially during the early stages of language development (Meara 1995; Chen and Chun 2008; Ali et al. 2012; Hasegawa et al. 2015). Yet learners also require consistent, meaningful language interaction. In the context of European Higher Education language learners face an increasing focus on independent learning using virtual platforms like Moodle, Blackboard, etc.—at the expense of in-class, face-to-face learning hours (Berns et al. 2013a; European Commission/EACEA/Eurydice 2015; Bates 2015). As a result, the focus still tends to be more on learning about a language than on learning to use the language as a vehicle for effective communication (Spada 1997; Berns et al. 2013b); learners are not given enough opportunities to interact and negotiate in the target language—essential to successful language acquisition (Swain and Lapkin 1995; Long 1996; Warschauer 1997; Gass and Mackey 2007). Hence, conventional teaching/learning approaches alone often fail to meet basic learning needs.

In recent years mobile learning has gained in popularity becoming a valid approach to complement traditional teaching/learning processes (Sánchez-Prieto et al. 2013). However, while the potential for using game-based and gamified apps to go beyond conventional approaches has already been explored to some extent in other areas such as Physical Education and Geography (Facer et al. 2004; Ly et al. 2012; Monguillot et al. 2014) in the area of language language learning few attempts have been made to explore such opportunities (Liu 2009; Al-Shehri 2011). A review of the literature has shown that despite the numerous attempts to incorporate technology in learning processes, using RFID tags (Ogata et al. 2010), interactive television (Fallakhair et al. 2007) and mobile phones (Petersen et al. 2009; Pemberton and Winter 2012), most apps fail to go beyond conventional learning approaches to harness the full potential of the technology (Chinnery 2006; Kukulska-Hulme and Shield 2008; Burston 2013, 2014, 2015). Consequently, they do not effectively meet students’ needs as the focus tends to be exclusively on individual learning rather than providing students with collaborative tools, which foster interaction and negotiation in the target language. To date, hybrid, game-based apps—combining individual and collaborative learning tasks—have yet to be designed and implemented on a large scale (Burston 2015; Palomo-Duarte et al. 2016).

For the purposes of this study the authors propose the use of a specifically designed hybrid game-based app (*VocabTrainerA1*). Game-based learning refers to the process and practice of learning through games (Lilly and Warnes 2009). This is mostly done through the use of serious games combining both fun and entertainment with educational purposes (Bellotti et al. 2013). Nonetheless, with a view to increase students’ participation and to make the learning process more motivating (Marín Díaz 2015) *VocabTrainerA1* includes also features of gamified learning. Gamification is the application of game-design elements and game principles in non-game contexts (Deterding et al. 2011; Francisco-Aparicio et al. 2013). In line with this approach the individual learning tasks of the app require students to solve traditional exercises (multiple choice, fill in the gaps, etc.) which integrate game-design elements (points, levels, scores, randomly delivered content and a time limit) in order to make tasks more playful and game-like as well as to leverage people’s natural desire for mastery, achievement, etc. The differentiation between game-based learning and gamified learning through the use of serious games has been widely discussed in the literature by authors such as Oliveira and Petersen (2014).

In a context where language classrooms are plagued by high enrolment numbers and limited contact hours—hence, low exposure to the target language—we started exploring the possibility of providing learners with a hybrid game-based app for smart mobile devices. One of our main objectives was to make independent learning more motivating, boost perceived usefulness and added value, increase acceptance and sustainability, and enhance learning outcomes; in short, to better meet students’ language learning needs. By designing our own app we were able to tailor it specifically to the course syllabus and to students’ needs.

In line with flipped learning approaches (Bagby 2013), hybrid game-based apps place the focus on actively working through challenges—interacting and negotiating in the target language—rather than on decontextualized learning. The ubiquitous nature of such apps facilitates taking flipped approaches a step further—not only bringing the real world into the classroom but taking the classroom into the real world.

The *VocabTrainerA1* app—designed specifically for this study—aims to address this reality by going beyond conventional approaches to provide students with a hybrid, game-based learning tool, combining individual and collaborative learning tasks. Like the majority of available apps, *VocabTrainerA1* provides learners with valuable language input and the advantages of mobile learning (Burston 2013, 2015). What differentiates the app—hence students’ experiences and learning outcomes—is the synergy created by combining individual learning tasks with an engaging collaborative role-play, in which learners are challenged to negotiate in the target language and apply what they have learned to solve a murder mystery with their peers. By designing a hybrid game-based app that not only delivers randomly-generated multimedia exercises but also requires students to apply their language knowledge to meaningful communication tasks, the authors aimed to motivate learners and meet their needs for more language exposure and interaction with other speakers (Chapelle 1998). This approach is more in line with the current theories of foreign language learning which focus primarily on enabling students to communicate effectively in the target language (Sanders and Kenner 1983; Mitchell 1994; Spada 1997; Moeller and Catalano 2015).

We aim to find qualitative and quantitative data supporting the following hypotheses:

### **H1**

Students will be motivated by the use of a hybrid game-based app for learning purposes and will perceive a high degree of usefulness and added value.

### **H2**

Using a hybrid game-based app will have a positive impact on learning outcomes.

We hope to find evidence supporting the premise that hybrid game-based apps, like *VocabTrainerA1*—which seamlessly combine individual and collaborative learning—will enjoy high acceptance and motivate learners, stimulate perceived usefulness and added value, and meet the language learning needs of today’s language learners more than conventional and non-collaborative technology-based approaches. In terms of outcomes and sustainability we hope to find data indicating that, for today’s digital natives (Prensky 2001; Bates 2015), hybrid game-based apps are, indeed, effective tools for language learning.

## Experimental setting

The present paper focuses on a case study using *VocabTrainerA1*—a hybrid game-based app for smart mobile devices—during a 4-week period with 104 beginner German language students (A1.2 CEFR) at a Spanish university. The purpose of our study was twofold: on one hand, to measure learner motivation and the degree of perceived usefulness and added value after using the *VocabTrainerA1* app; on the other, to measure the impact of using a hybrid game-based app on learning outcomes. Hence, the following hypotheses:

### **H1**

Students will be motivated by the use of a hybrid game-based app for learning purposes and will perceive a high degree of usefulness and added value.

### **H2**

Using a hybrid game-based app will have a positive impact on learning outcomes.

The experiment was carried out during the second semester of the 2013–2014 academic year. In their first semester German language course (A1.1 level, CEFR) students had used the university’s Moodle platform to access learning resources (audio, video, clozes, forums, etc.). However, apps for mobile devices had never been employed.

The data which have been used for the current case study were obtained from different sources. These sources include students’ grades from the first semester, based on several writing tasks as well as the data from the second semester, based on different tests and the interactions stored by the game server when using the *VocabTrainerA1* app. Being one of the authors the tutor of both language courses (first and second semester) the information was available under proper confidential restrictions. The information was used to check if there was any correlation between students’ learning outcomes before and after using the app.

In order to familiarise students with the *VocabTrainer A1* app, a 1-h training session was held during which students were asked to perform as many game tasks as possible. Over the next 2 weeks students were encouraged to play the individual part of the app on their own in order to complete and repeat all game tasks. Game repetition was aimed at reinforcing vocabulary, grammar, reading and writing skills, in preparation for the collaborative game task. Only after successfully completing the individual tasks were students allowed to participate in the collaborative game task—a murder mystery game, called *Catch Me, If You Can!* in which students were expected to apply previously acquired language skills to real-life communication.

**Fig. 1:**
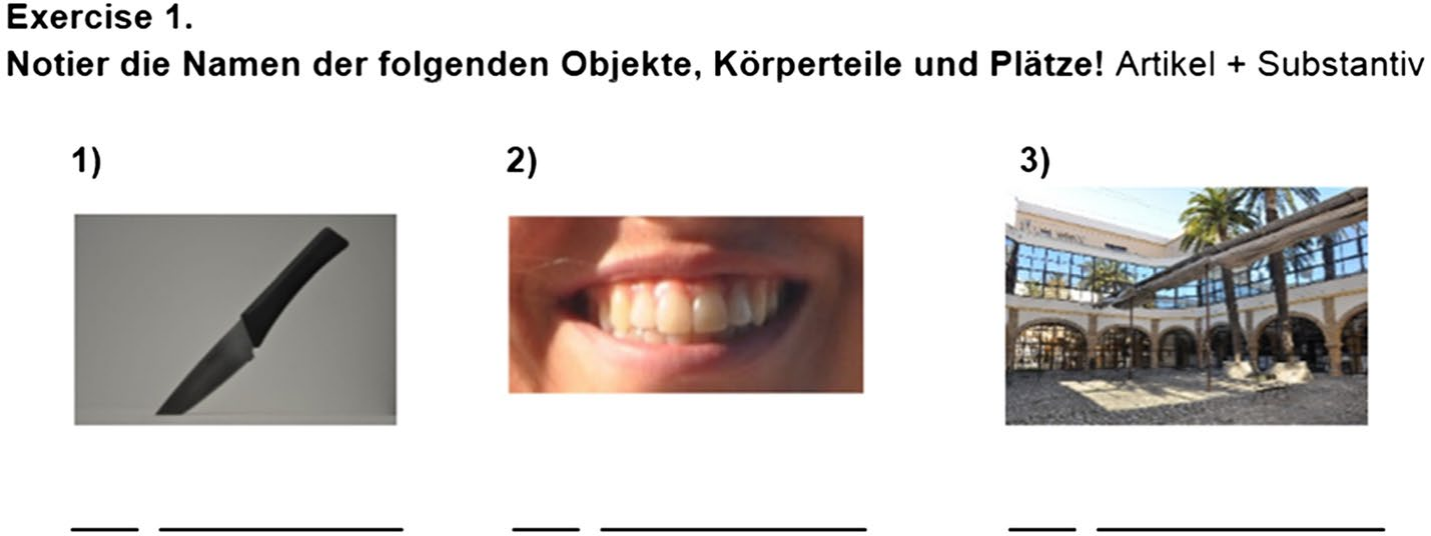
Exercise 1 focuses on the use of nouns (vocabulary) and their respective articles (grammar)

**Fig. 2:**
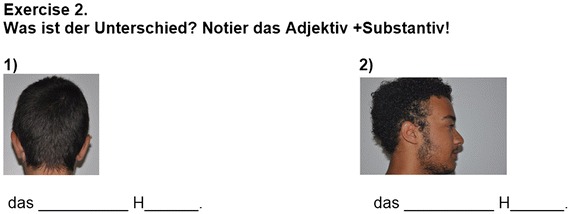
Exercise 2 focuses on the use of nouns (vocabulary) and their respective adjectives (grammar)

**Fig. 3:**
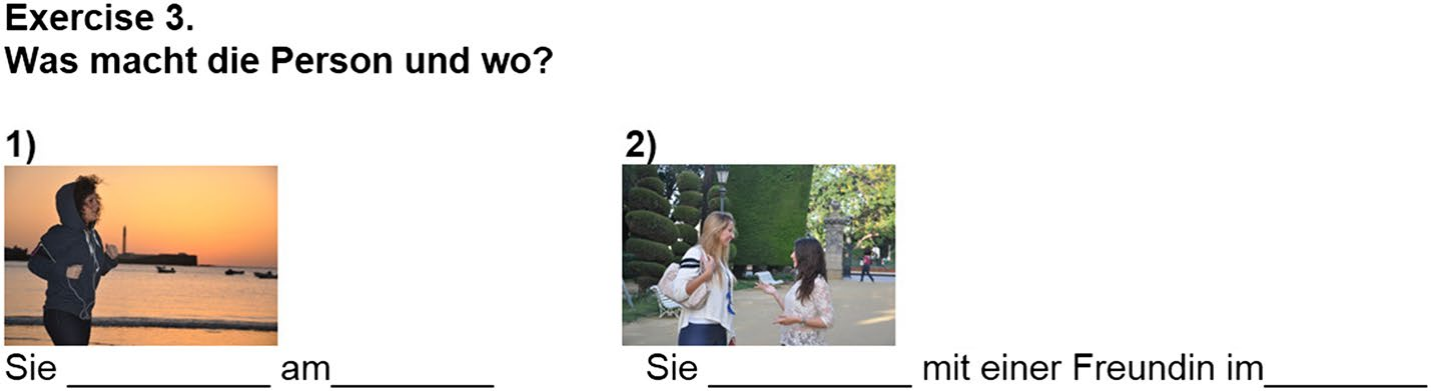
Exercise 3 focuses on indicating actions (grammar) and where they take place (vocabulary)

To measure the impact of the app on language learning, students were asked to take a pre-test—prior to using the app—and a post-test, immediately following the experiment. Both tests were designed to evaluate students’ grasp of the language skills covered by the *VocabTrainerA1* app. The tests contained 50 questions each, divided into three exercises (see Figs. 1, 2, 3). However, the vocabulary and grammar items on each test were selected randomly. In doing so, the authors aimed firstly, to guarantee that the entire content of the app was tested and secondly, that students had to focus on all items during the learning process.

An anonymous Technology Acceptance Model survey (TAM) and focus group interviews were conducted to measure learner motivation and the degree of perceived usefulness and added value after using the *VocabTrainerA1* app. In addition, we aimed to determine acceptance and sustainability of our hybrid game-based app in the short to mid-term (see Appendix 1, Table 7). The survey was designed in line with the model proposed first by Davis (1989) and revised by Liu et al. (2010). The Likert-based survey—ranging from 1 (minimum) to 5 (maximum)—was conducted with 91 students out of the total sample population of 104.

With a view to gather more detailed feedback on students’ individual experiences—and eventually revise and enhance the app—we conducted a series of focus group interviews (Krueger and Casey 2010). Interviews were designed in line with the theoretical approach of Interpretative Phenomenological Analysis (IPA) and aimed at encouraging “a reflective engagement with the participant’s account” focusing on the participants’ experience and the meaning they make of the experience (Smith et al. 2009). Data have been first gathered through several interviews (see Appendix 2, Table 8) analysed under different themes in line with the topics addressed within the interviews (motivation, usefulness, effectiveness, efficiency). Interviews were carried out with a sample population of 12 students with different learning profiles, divided into three groups. Each group interview lasted approximately 60 min. To make students feel more at ease when expressing their personal opinions about the app, we asked two external supervisors to carry out the interviews.

### Game design

*VocabTrainerA1* is a two-part hybrid game-based app. The first part (Levels 1–3) is focused on individual learning and offers a number of offline game tasks. The second part (Level 4) is an online collaborative murder mystery game, called *Catch Me, If You Can!*, in which students must work together to identify a serial killer (see Table 1).

**Table 1:** Game structure and content

Levels	Play-modes	Topics and vocabulary	Grammar	Language Skills	Tasks
1–3	Individual (offline) Against time-limit	Places and activities	Articles	Listening Listening and Reading Reading and writing	Identifying places Matching places with their names Describing places and activities
		Physical features and personal objects	Adjectives	Listening Listening and reading Reading and writing	Identifying personal objects and physical features Matching personal objects and physical features with their names Describing personal objects and physical features
		Body parts and personal characteristics	Verbs	Listening Listening and reading Reading and writing	Identifying body parts Matching body parts with their names Describing a person’s body parts and personal characteristics
4	Collaborative (online) In teams	Witnesses and the serial killer	Articles, verbs and adjectives	Reading, listening and writing	Identifying witnesses and catching the serial killer

As can be seen in Table 1, each level focuses on different topics, vocabulary and grammar, providing opportunities to practice a variety of language skills (listening, reading and writing) through the completion of specific tasks. Levels 1 through 3 aim to provide individual learners with vocabulary and grammar input. Level 4—which can only be played after successfully completing Levels 1 through 3—takes learning a step further by requiring students to apply their language skills in real-world-like communication with peers. It is the synergy created by combining individual learning tasks (Levels 1–3) with an engaging collaborative role-play (Level 4) which makes *VocabTrainerA1* a hybrid game-based app—and differentiates it from the majority of language learning apps on the market (Berns and Palomo-Duarte 2015).

### Individual game-task design

In the individual learning phase (Levels 1–3) the app delivers both instant and delayed feedback. Delayed feedback goes beyond whether student responses are correct or incorrect, providing learners with detailed information on their performance, along with a range of possible answers (Sun et al. 2008). This type of feedback becomes especially important at Level 3 where tasks are more complex and players are required to write in the target language. Tasks are designed to be both engaging and challenging—but also more effective in terms of learning outcomes—through the integration of game features such as levels, a scoring system, randomly delivered content and a time limit. Finally, students must score 90 % or higher in order to move on to the next level. This feature fosters learning through game-repetition (Berns and Palomo-Duarte 2015; Hasegawa et al. 2015).

### Collaborative game-task design, setup and play

In the collaborative role play (Level 4), learners work together in teams of three to identify a serial killer, who is planning another crime. Each team consists of two roles: one detective and two police officers. At least one team is required but there is no limit—other than server capacity—on the number of teams which can play concurrently (Berns and Palomo-Duarte 2015). Learners play for 1 h, allowing each team several opportunities to identify different killers.

**Fig. 4:**
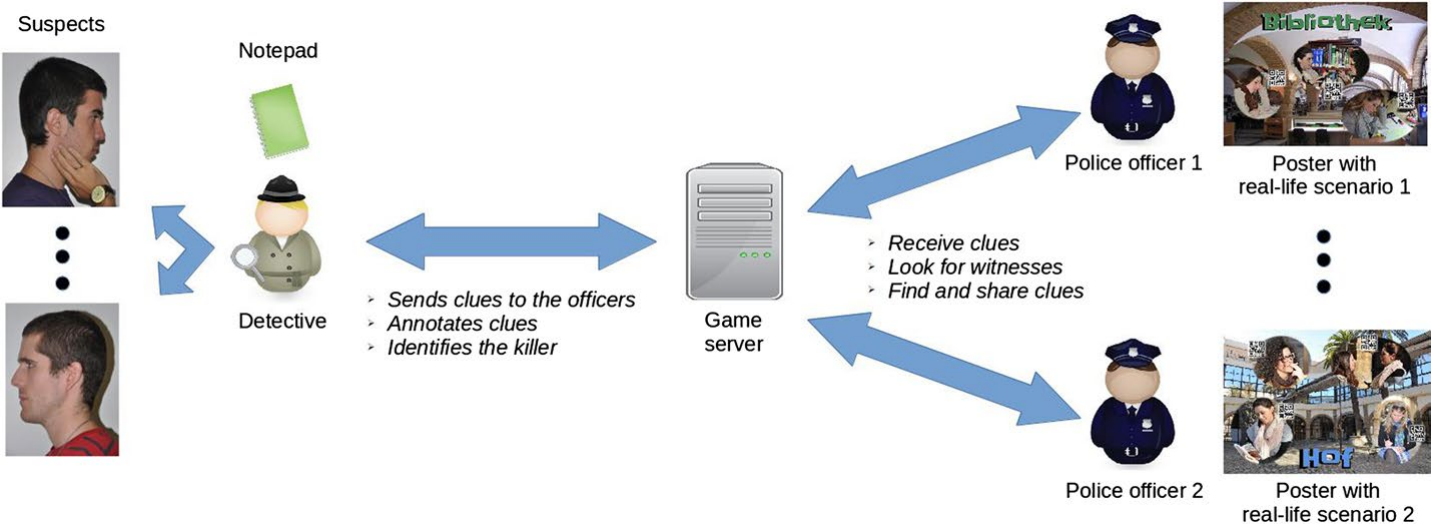
Roles and tasks

Prior to starting play, the supervisor must prepare a distinct playing field for each role: one for the detectives and another for the police officers. For the detectives, a room must be set up with a separate poster for each of the 24 different suspects (see Fig. 4). For the police officers, QR codes linked to short video-clips of witnesses providing clues are superimposed on posters depicting a variety of real-life scenarios (a supermarket, a parking lot, a library, etc.). These posters are placed randomly around the playing field (e.g. in different places of the university building). Finally, the supervisor must indicate the different teams to the server.

Once the playing fields have been set up, each player is automatically assigned a role: detective or police officer. Detectives are given the task of coordinating the investigation via the anonymous in-app text chat of their group—and of eventually identifying the killer. Police officers must identify the witnesses and provide detectives with information. Play starts with each role in their playing field. Detectives receive two clues from the server in the target language on how and where to find the first two witnesses. These clues must be shared with the officers via the in-app text chat^1^ (see Fig. 4).

**Fig. 5:**
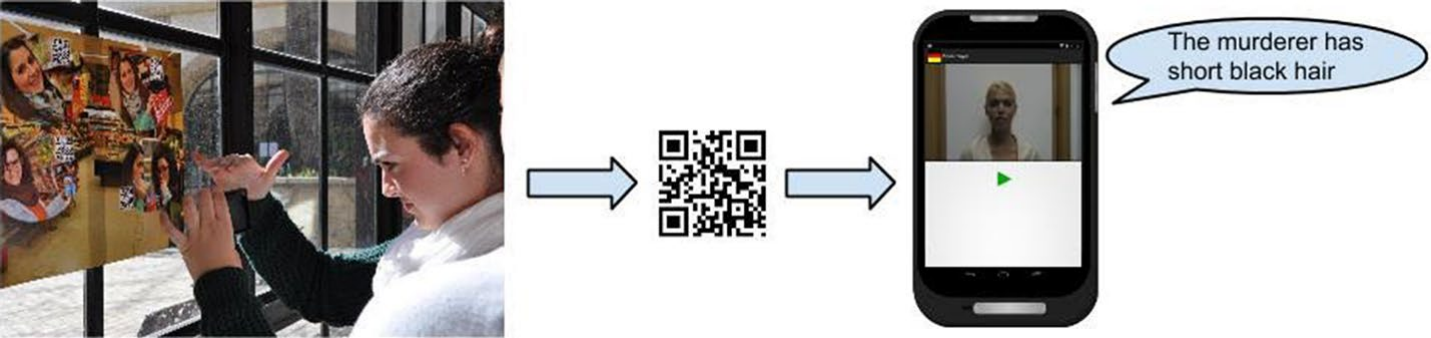
A police officer scanning a QR code and a video-clip of witness delivering information

Police officers are expected to use the clues to identify two witnesses on the posters placed around the playing field. Once one of the witnesses has been identified, officers must scan the QR code and watch a short video-clip, in which the witness provides clues about the serial killer (see Fig. 5).

One of the police officers then sends this information to the other officer and the detective, who must annotate it via the in-app notepad (see Fig. 4). These annotations are fundamental since they will help the detective identify the serial killer. Once an officer has identified a witness and scanned the corresponding QR code, all team members are automatically notified by the system and a new clue is sent to the detective.

Play continues until detectives have enough information to identify the killer. To do so the QR-code on the suspect poster in the detectives’ playing field must be scanned, at which time all team members are notified by the system and the game ends.

The game contains 24 different clues which lead to different serial killers. Clues are displayed randomly, allowing players to repeat the game several times—each time facing a new challenge. The app has been designed so as to allow the teacher to easily modify and/or increase the number of clues and killers. The following table shows a simplified chat (see Table 2).

**Table 2:** Fragment from an in-app chat conversation

Sample player actions	Sample verbatim chat interaction
The Detective logs in and launches the game The server automatically sends 2 clues to the Detective regarding the location of the first two witnesses **Examples** Clue 1: *Witness 1 is at the lake talking on the phone* Clue 2: *Witness 2 is at the garage paying for the parking*	[14:49] Server: ***Kommissar ist online*** (Server: *The Detective is online.)*
Police Officer 1 logs in and joins the game	[14:50] Server: ***Polizist 1 ist online*** (Server: *Police Officer 1 is online.*)
Police Officer 2 logs in and joins the game	[14:50] Server: ***Polizist 2 ist online*** (Server: *Police Officer 2 is online.*)
The Detective enters Clue 1 and shares it with the Police Officers	[14:50] Kommissar: ***Zeuge 1 ist im See und telefoniert*** (Detective: *Witness 1 is at the lake and talking on the phone*)
Police Officer 1 communicates he/she will investigate Clue 1 and looks for the location (*the lake*)	[14:51] Polizist 1: ***ich gehe*** [sic] (Police Officer 1: *i’ll go*) [sic]
The Detective enters part of Clue 2 and shares it with the Police Officers	[14:51] Kommissar: ***Zeuge 2 ist im Tiefgarage*** [sic] (Detective: *Witness 2 is in carpark*) [sic]
Police Officer 2 communicates he/she will investigate and looks for the location (*the carpark*)	[14:51] Polizist 2: ***ich gehe*** [sic] (Police Officer 2: *i’ll go*) [sic]
The Detective provides the officers with more details regarding the Clue 2	[14:51] Kommissar: ***…und zahlt*** (Detective:*…paying*)
Police Officer 1 uses the first clue to identify the witness and scans the QR code	[14:51] Server: ***Polizist 1 hat QR code gescannt*** (Server: *Police Officer 1 has scanned the QR code.*)
The server automatically confirms that the QR code is correct and delivers a video-clip providing meaningful information about the killer to the officer who has scanned the code	[14:51] Server: ***Ja, du hast Zeuge 2 gefunden*** (Server: *Yes, you have identified Witness 2.*)
Police Officer 2 uses the second clue to identify the witness and scans the QR code	[14:52] Polizist 2: ***scannt QR code*** (Server: Police Officer 2 *has scanned the QR code*)
The server automatically confirms that the QR code is correct and delivers a video-clip providing meaningful information about the killer to Officer 2	[14:52] Server: ***Ja, du hast Zeuge 1 gefunden*** (Server: *I’ll go.*)
Police Officer 1 enters the information about the killer from the video-clip and shares it with the Detective and Officer 2	[14:52] Polizist 1: ***Zeuge 2 hat kurzes Haar*** (Police Officer 1: *Witness 2 has short hair*)
Police Officer 2 enters the information about the killer from the video-clip and shares it with the Detective and Officer 1	[14:52] Polizist 2: ***Zeuge 1 ist 20 jahre alt*** [sic] (Police Officer 2: *Witness 1 is 20 Years old*) [sic]
The server automatically sends 2 new clues to the Detective regarding the location of two new witnesses **Examples** Clue 3: *Witness 3 is reading in the courtyard* Clue 4: *Witness 4 is chatting on the beach* The Detective enters Clue 4 (*Witness 4 is chatting on the beach)* and shares it with the Police Officers	[14:52] Kommissar: ***zeuge 3 ist im Hof und liest*** [sic] (Detective: *witness 3 is in the courtyard, reading*) [sic]
Police Officer 2 communicates he/she will investigate Clue 3 and looks for the location (*the courtyard*)	[14:52] Polizist 2: ***ich gehe*** [sic] (Police Officer 2: *i’ll go*) [sic]
The Detective enters Clue 3 and shares it with the Police Officers	[14:53] Kommissar: ***zeuge 4 ist am Strand und plaudert*** [sic] (Detective: *witness 4 is chatting at the beach*) [sic]
Police Officer 1 communicates he/she will investigate Clue 4 and looks for the location (*the beach*)	[14:53] Polizist 1: ***ich gehe*** [sic] (Police Officer 1: *i’ll go*) [sic]
(…)	(…)
The server informs the players that all 12 witnesses have been identified	[15:03] Server: ***Ja, du hast Zeuge 12 gefunden*** (Server: *Yes, you have identified Witness 12.*)
The Detective still needs more information to identify the killer and asks the officers if the suspect has a weapon	[15:03] Kommissar: ***keine Tatwaffe?*** [sic] (Detective: *no* *murder weapon?* [sic]
Police Officer 2 doesn’t understand the German word for weapon and asks for clarification	[15:04] Polizist 2: ***was ist das?*** [sic] (Police Officer 2: *what’s that?*) [sic]
Police Officer 1 doesn’t understand either and asks what “Tatwaffe” means	[15:04] Polizist 1: ***was is Tatwaffe?*** [sic] (Police Officer 1: *what is murder weapon?*) [sic]
The Detective clarifies what “Tatwaffe” means by providing examples of different types of weapons in German	[15:05] Kommissar: ***Schere, Beil, Messer*** (Detective: *Scissors, hatchet, knife*)
Police Officer 1 is still confused as well	[15:06] Polizist 1: ***ich nicht hört das*** [sic] (Police Officer 1: *i don’t hear that*) [sic]
The Detective decides to take a guess and scans one of the QR codes on the suspect profile posters	[15:06] Kommissar: ***Mm*** [sic] (Detective: *Mmm*) [sic]
The server confirms that the Detective not identified the serial killer	[15*:06]* Server: ***Der Kommissar hat nicht den Mörder identifiziert*** (Server: *The Detective hasn’t identified the killer*)

### Game architecture

*VocabTrainer A1*—a game-based language-learning app with a hybrid, level-based architecture—is freely available for Android devices and can be installed from the download section of its public forge.^2^ The app contains all the multimedia needed for the different game-tasks (audio recordings, video-clips, photos, etc.) and an in-app text chat function.

**Fig. 6:**
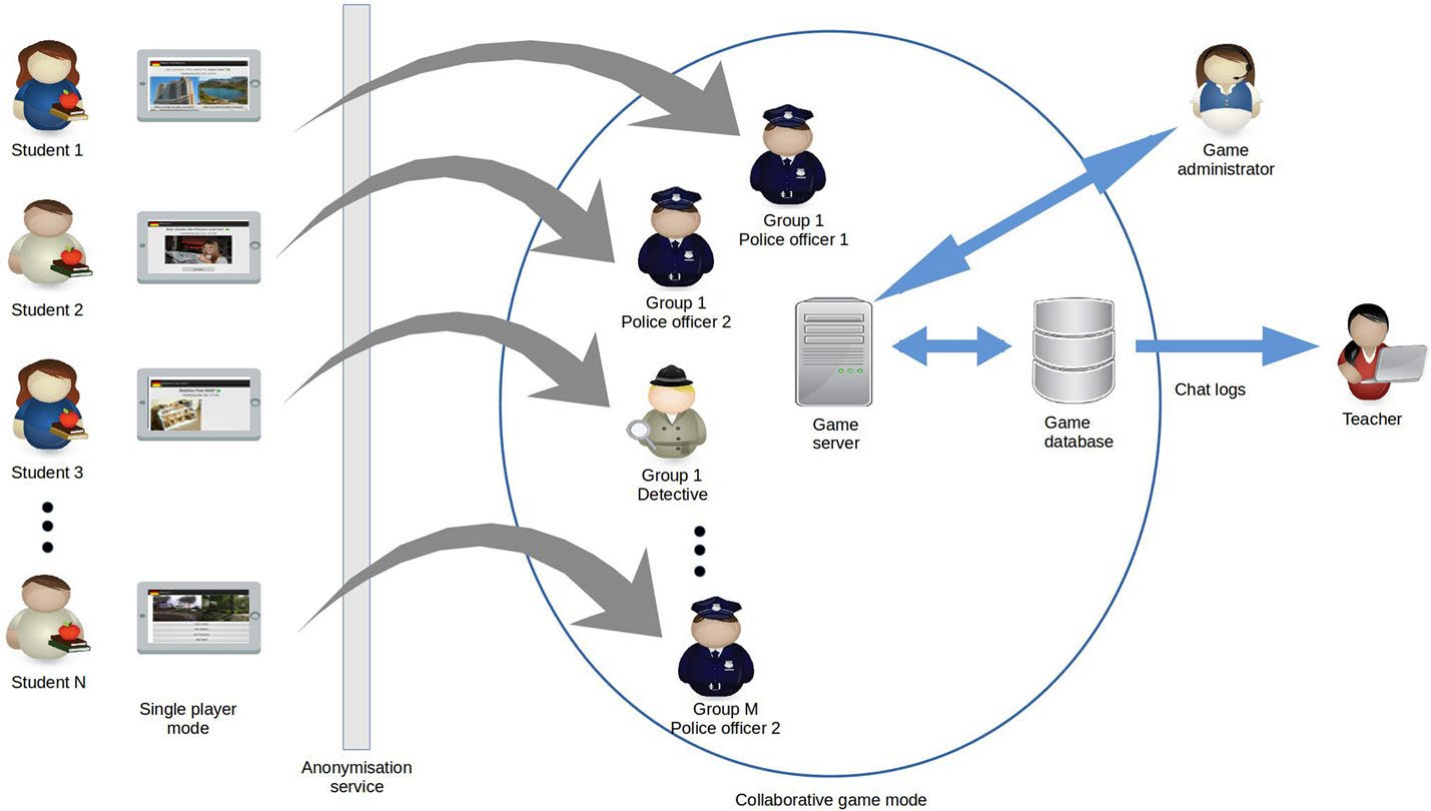
Game architecture

*VocabTrainer A1* was designed using two distinct architectures: whilst the individual learning tasks (Levels 1–3) are played locally on students’ mobile devices, the collaborative task (Level 4) can be played only by connecting the app to a properly configured server. Once learners have successfully completed the first three levels, a server-generated username and password are provided to access Level 4. At this point, the app connects to the server and the game administrator can launch the role play task. We have used the Openfire server which allows for 3-way, real-time in-app communication between the system and the players (see Fig. 6). As Level 4 is played online, the server must be properly configured to accept connections from students’ mobile devices.

To prevent students from communicating with their team members via channels other than the in-app text chat, the server generates anonymous player identities. The basic functions of the server include: assigning teams, delivering clues to detectives, displaying video-clips to police officers, providing ongoing feedback to students regarding correct/incorrect responses and terminating the game once the killer has been identified.

## Discussion and evaluation

With a view to provide both qualitative and quantitative evidence supporting our initial hypotheses, in this section we will analyse data from the TAM survey and focus group interviews conducted after using the *VocabTrainerA1* hybrid game-based app. We will also analyse learning outcomes at two different stages: on the one hand, performance on first-semester conventional writing tasks as compared to results on the pre-test—both prior to using the app; on the other, performance on the post-test, taken immediately after using the app. In addition, scores obtained in the conventional writing tasks, the pre-test and the post-test are analysed to determine whether any correlation exists. All data seem to support our original premise, that the combination of individual and collaborative learning tasks—in a hybrid, level-based architecture—motivates and meets students’ needs more than conventional learning approaches, and has a positive impact on learning outcomes.

### Qualitative analysis

#### Technology acceptance model survey (TAM) and focus group interviews

The literature indicates the need for target users to be integrated in the design process in order to ensure both that learners are motivated to use the tool and that learning needs are met (Nelson and Oliver 1999; Kennedy and Levy 2009). In line with this approach our TAM survey focuses exclusively on the experiences of learners and their evaluation of the app as a language learning tool. Motivation, usefulness and added value are measured by gathering direct feedback from students regarding their experience with the app, as part of a 3-phase iterative development process (experimentation-evaluation-enhancement) requiring the involvement of the learners themselves (Kennedy and Levy 2008). As all answers were provided anonymously they cannot be correlated with scores. However, the low degree of deviation indicates that the mean is a fairly accurate indicator of opinions across the entire population (see Appendix 1, Table 7).

Results from the TAM survey support Hypothesis 1, indicating that learners are indeed motivated by the use of hybrid game-based apps for learning purposes and perceive a high degree of usefulness and added value (see Appendix 1, Table 7). In a range from 1 to 5, students reported that app content was interesting (4.47 points; Std. dev. 0.70) and met their learning needs (4.32 points; Std. dev. 0.82). The vast majority of learners found the on-going feedback the app provides to be very useful (4.51 points; Std. dev. 0.80). Furthermore, learners confirmed that the app motivated and helped them to improve key language skills such as reading (4.27 points; Std. dev. 0.81), writing (4.51 points; Std. dev. 0.67) and vocabulary (4.83 points; Std. 0.48)—and enhanced their overall linguistic competence (4.55 points; Std. 0.68) and fluency (4.26 points; Std. dev. 0.48). In all cases the low degree of deviation indicates that these results are a fairly accurate indicator of perceptions across the entire population.

Especially noteworthy is the high value students place on the app, as a tool for learning vocabulary (4.83 points; Std. dev. 0.48). This perception is reinforced by comments made during the focus group interviews—which also provide qualitative evidence supporting Hypothesis 1.^3^

- ST1: It was very useful. The app helped me learn tons of vocabulary. It’s not the same to memorise a definition as to make a mental image of what you’re trying to learn.
- ST2: It was a completely different way of learning vocabulary.
- ST3: You learned by playing, and you learned more. It wasn’t like sitting down and studying—much more exciting. It was really entertaining and I played every chance I got!

Here students highlight that using the app was not only more effective, but more engaging and fun than conventional learning tools such as wordlists, flashcards and clozes. This is far from trivial. Fun learning is motivated learning—and often leads to better outcomes.

- ST4: The images motivated me very much and made learning very practical and fun. It’s a better way of learning. The time limit is like a personal challenge and is good for prepping for the exam, where you don’t have much time.
- ST5: It’s a very competitive app, that’s what motivated me. The time limit and different game levels pushed me to challenge myself and improve. I could actually see the results of my efforts, and being able to track my progress was very useful. I was hooked until the end—which helped me to learn a lot very quickly.
- ST6: I had a lot of fun competing. It was a new way of learning—much more addictive than the traditional way, using books, the virtual campus and all that. I just loved the role play. I couldn’t stop playing it—it’s so fun to communicate with classmates in a new language and learn by doing!

In the interviews, learners return again and again to the features they found most motivating: game features such as a scoring system, time limits, play levels and multimedia content such as photos, video and audio made the app more practical, challenging and fun than conventional learning tools. Students seem to agree that the interactive, competitive nature of the app and its anonymous text-chat brought learning to life.

- ST7: The role play was cool, really interactive. It was especially useful because we had to apply what we’d already learned to communicate with our mates, which was a good laugh!
- ST8: The role play helped me learn to communicate much better! In the first levels of the app you learn what words mean, you memorise them and learn to add the article. The role play was a great way to review all that. Plus, since nobody’s looking, it’s not so embarrassing!
- ST9: The role play helped me loosen up and stop being terrified of making mistakes. The text chat is private; it’s anonymous and no one’s watching you. Looking back, I think it was mainly the chat which helped me lose my fear of making a fool of myself, when I started to communicate more fluently.

Learners clearly indicate that having to apply their language knowledge in the collaborative role play at Level 4 (*Catch Me, If You Can!*) contributes very positively to the added value of the app. Furthermore, results from the TAM survey indicate that learners place high value on the opportunities the app affords for communicating and negotiating in the target language (4.65 points; Std. dev. 0.55)—opportunities which are very rare when using conventional learning tools. This is seen as being especially valuable for improving fluency (4.26 points; Std. dev. 0.48).

Yet another area where added value is perceived is the ubiquitous nature of the app. Learners appreciate the flexibility and comfort apps afford, along with the opportunity to streamline time management by making the most of commutes, etc. In the same way that multimedia and virtual reality can bring real-world scenarios into the classroom, smart mobile devices take the classroom into the real-world. Furthermore, students indicate that their positive experience with the app has piqued their interest in continuing to use this kind of tool for language learning.

- ST10: Learning with my mobile phone was great, much more comfortable. You can study anywhere; no need to lug heavy books around or anything like that. I used to play on the train a lot, I loved it! Mobile phones are good because you can play whenever you want.
- ST11: I really enjoyed the role play. I’ve always been a fan of learning through games. So after having so much fun playing the app, I started browsing the internet and going on Twitter to try and find more language apps to play. Unfortunately, it was quite hard to find good apps.
- ST12: The app had a big impact on me because I’d never used an app to learn a language before. In fact, thanks to this experience, I now have several apps in my phone which I wouldn’t have otherwise. I’d love it if there were more apps like this for learning German, and other languages too!

Students express a desire to continue using hybrid game-based apps for language learning, and that using *VocabTrainerA1* has motivated them to take finding new apps into their own hands. This is corroborated by the results from the TAM survey. The vast majority of students confirm that they intend to use apps like *VocabTrainerA1* more often to improve their language proficiency in German and other languages (4.51 points; Std. dev. 0.60).

This suggests that hybrid game-based apps designed with students’ interests and learning needs in mind are sustainable in the long term—in line with Nelson and Oliver (1999) and Kennedy and Levy (2009).

### Quantitative analysis

Thus far, discussion has centred on qualitative analysis providing evidence in support of

Hypothesis 1: *Students will be motivated by the use of a hybrid game*-*based app for learning purposes and will perceive a high degree of usefulness and added value*. In this section attention is turned to quantitative analysis providing evidence in support of Hypothesis 2: *Using a hybrid game*-*based app will have a positive impact on learning outcomes.*

### Pre-test versus post-test scores

In line with our initial hypothesis (H2), pre-test and post-test scores indicate that learning outcomes improved significantly after using *VocabTrainerA1* (see Table 3).

**Table 3:** Average pre-test and post-test scores

	Maximum score	Pre-test Average	Pre-test Std dev	Post-test average	Post-test Std dev	Average gain	Gain Std. dev.
Pre-test/post-test total	100	18.38	10.92	75.52	14.42	57.14	13.15
Vocabulary total	50	9.49	5.83	42.76	6.82	33.27	6.82
Grammar total	50	8.89	5.70	32.76	8.86	23.87	7.99
Exercise 1 total	20	2.77	2.16	15.26	3.71	12.49	3.65
Exercise 1.V	10	1.33	1.11	8.47	1.59	7.13	1.59
Exercise 1.G	10	1.44	1.20	6.79	2.34	5.35	2.35
Exercise 2 total	20	4.04	2.97	10.92	3.64	6.88	3.76
Exercise 2.V	10	3.45	2.41	8.07	2.76	4.62	3.51
Exercise 2.G	10	0.59	1.46	2.86	2.99	2.27	2.72
Exercise 3 total	20	6.03	3.25	18.82	2.53	12.79	3.95
Exercise 3.V	10	2.04	1.59	9.29	1.37	7.25	1.99
Exercise 3.G	10	3.99	2.20	9.53	1.31	5.54	2.45

The overall student average of 18.38 points in the pre-test (Std. dev. 10.92) went up to an average of 75.52 in the post-test (Std. dev. 14.42)—an average gain of 57.14 points (Std. dev. 13.15).

**Fig. 7:**
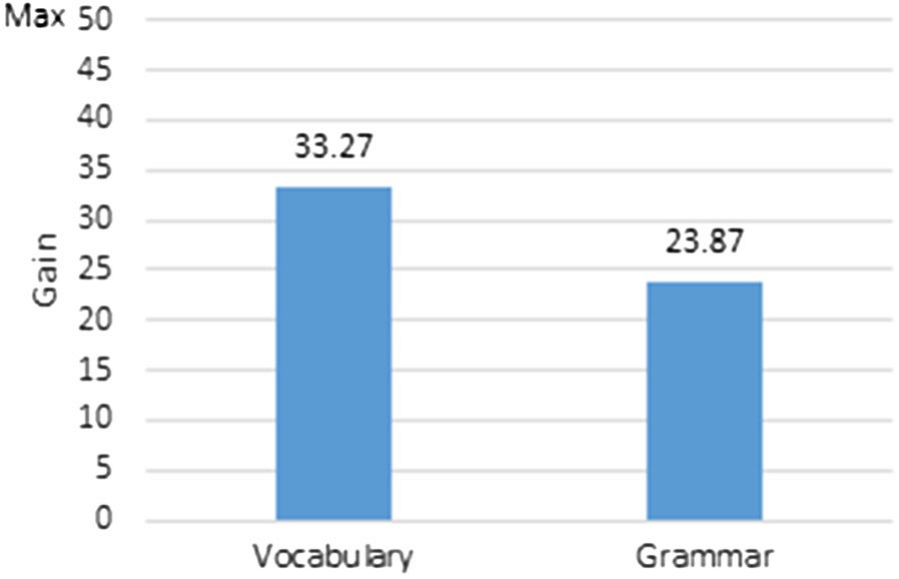
Average vocabulary versus grammar gain

A deeper look at student performance with regard to different linguistic aspects, however, provides greater insight into learning outcomes. The figures from Table 3 indicate that, when using the app, students benefited more in terms of vocabulary than in terms of grammar. The average pre-test score for grammar was 8.89 (Std. dev. 5.70), rising to a score of 32.76 (Std. dev. 8.86) in the post-test. In contrast, the average pre-test score for vocabulary was 9.49 (Std. dev. 5.83), rising to 42.76 (Std. dev. 6.82) in the post-test. Thus the average gain for grammar was only 23.87 points (Std. dev. 7.99) versus a more homogeneous gain of 33.27 points (Std. dev. 6.82) for vocabulary—a gain of almost 10 points more (see Fig. 7).

**Fig. 8:**
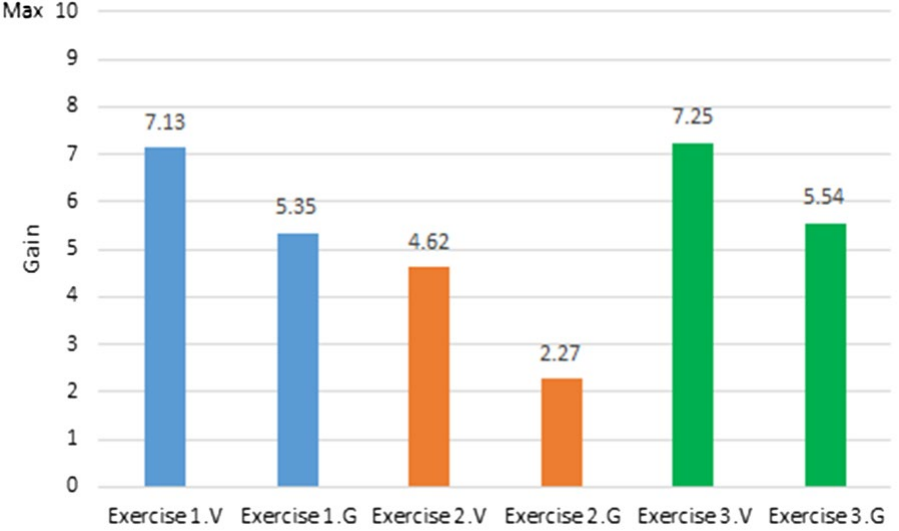
Vocabulary and grammar gains across exercises

Despite gains in vocabulary scores being higher than gains in grammar scores, a comparative analysis of results for Exercises 1, 2 and 3 indicates that learning outcomes were not uniform across exercises for either vocabulary or grammar (see Fig. 8).

In Exercise 1, where students were asked to indicate different nouns (vocabulary) and their corresponding article (grammar), vocabulary scores improved by 7.13 points; grammar scores improved by 5.35 points. In Exercise 2, students had to indicate different nouns (vocabulary) and their corresponding adjectives (grammar). In this case, however, the average gain for vocabulary was only 4.62 points—a gain of 2.51 less than in Exercise 1. Similarly, the average gain for grammar was only 2.27 points, a gain of 3.08 points less. In Exercise 3, students were asked to indicate an action (grammar) and where it took place (vocabulary). In this case vocabulary scores improved by 7.25 points and grammar scores improved by 5.54 points—similar gains as those in Exercise 1.

The significantly lower gain obtained in Exercise 2—with respect to Exercises 1 and 3—is likely due to a basic linguistic difference between Spanish, the students’ mother tongue, and German, the target language. While in both languages adjective endings are usually determined by noun gender and number, in German there are three gender cases (masculine, feminine and neutral) while in Spanish there are only two (masculine and feminine). This aspect of German grammar usually requires extensive practice on the part of learners. However, learning outcomes were significantly improved in a much shorter period of time when using the hybrid game-based app.

A one-tailed paired-sample *T* test was carried out in order to compare the mean pre-test score with the mean post-test score. Tables 4 and 5 show the results obtained when performing an SPSS analysis of the data:

**Table 4:** Paired-sample statistics

	Mean	N	Std. Deviation	Std. Error mean
Pair Pre-test	18.38	104	10.920	1.071
Post-test	75.52	104	14.417	1.414

**Table 5:** Paired-sample test

	Paired differences			95 % confidence interval of the difference		t	df	Sig. (2-tailed)
	Mean	Std. Deviation	Std. error mean	Lower	Upper			
Pair Pre-test—Post-test	−57.13	13.14	1.28	−59.69	−54.57	−44.31	103	.000

The data indicate—at 95 % confidence level—that learning outcomes are lower, prior to using the app. Improvement in student performance oscillates between 54.578 and 59.691 points, as reflected in mean test scores.

### Correlation between conventional writing task and pre-test scores

**Fig. 9:**
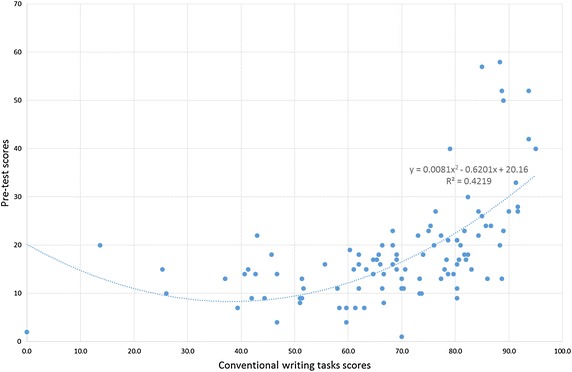
Correlation between conventional writing task and pre-test scores

In this section, conventional writing task and pre-test scores obtained prior to using *VocabTrainerA1* are analysed to determine whether any correlation exists. Contrary to our initial assumption—that there would be a linear correlation across the sample population—the data indicate the existence of a limited polynomial correlation ( see Fig. 9).

Students who obtained low scores in conventional writing tasks also performed poorly in the pre-test; likewise a small percentage of students with high scores in conventional writing tasks also performed well in the pre-test. Hence, in both cases, there is a correlation between conventional writing task and pre-test scores. In contrast, students who obtained mid-range scores in conventional writing tasks—and the majority of students with high scores—performed poorly in the pre-test, showing no correlation.

### Conventional writing task versus post-test scores

As previously observed when comparing outcomes for the pre-test and the post-test, an analysis of conventional writing task and post-test scores shows that a majority of students (65.05 %) obtained higher learning outcomes after using *VocabTrainerA1* (see Table 6).

**Table 6:** Average conventional writing task versus post-test scores

Conventional writing task average	Conventional writing task Std. Dev.	Post-test average	Post-test Std. Dev.	Average Gain
68.57	17.89	75.52	14.42	6.95

The overall average of 68.57 points in the conventional writing tasks (Std. dev. 17.89) went up to an average of 75.52 in the post-test (Std. dev. 14.42). Hence there is an average gain of 6.95 points—providing further evidence in support of Hypothesis2.

### Correlation between conventional writing task scores and relative learning gain

For the purposes of this study, we define relative learning gain as the ratio for score improvement (pre-test to post-test) for individual students.

$$ {\text{Relative}}\,{\text{learning}}\,{\text{gain}} = \left( {{\text{PostTestScore}} - {\text{PreTestScore}}} \right)/\left( {100 - {\text{PreTestScore}}} \right) $$

**Fig. 10:**
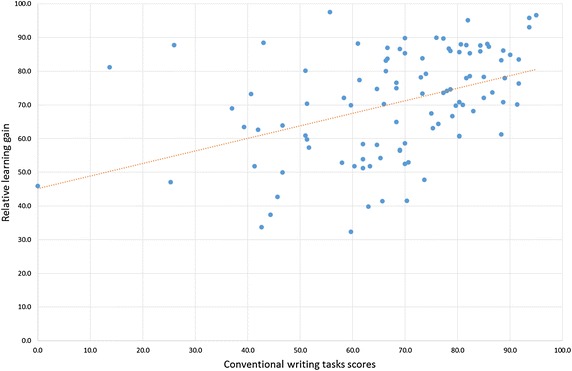
Correlation between conventional writing task scores and relative learning gain

The correlation coefficient between conventional writing task scores and the relative learning gain is 0.42, a moderate positive correlation. However, a closer look at the figures reveals two different performance patterns (see Fig. 10).

We can observe that—for students scoring 75 per cent or higher in conventional writing tasks—the standard deviation of the relative learning gain is 10.1. In contrast, the standard deviation among students scoring under 75 per cent is 16.4—significantly higher. This suggests that relative learning gain is more homogeneous for high-scoring students (≥75 %) than for students with mid-range and lower scores (<75 %).

Interestingly, a closer look at the data for students with mid-range and lower scores in conventional writing tasks (<75 %), again reveals two different performance patterns. The majority of the students in the group (74.58 %) shows a positive correlation (0.64) between the results obtained by this group in conventional writing tasks from the first semester and their relative learning gain. In contrast, the other 25.42 % of the students obtained a higher relative learning gain than the average of top students and their correlation coefficient is close to zero (−0.03), showing no linear correlation between them. This suggests that these students benefited from using *VocabTrainerA1* more than the rest of their peers.

### Threats to validity

In the current study different threats have been detected:

- Internal validity: in mid-term experiments like the one conducted for this study there are different aspects that may affect the results. During the 4 weeks lasting experiment students were exposed to additional language input both in class as well as outside class (virtual learning platform, etc.). Other variables may have influenced students’ learning outcomes, so that the authors cannot definitely confirm that the results obtained are solely due to the influence of the intervention. Further studies are needed to analyse the influence of other variables.
- External validity: Due to the relatively small and restricted sample size used for the present case study the results can be generalized only in the context of the course and the institution the results were obtained. A much larger and diversified sample size is needed to extrapolate data and draw stronger conclusions on the app’s validity to increase students’ motivation and learning outcomes.

## Conclusions

Vocabulary input is a primary learning need, especially during the early stages of language development (Meara 1995; Chen and Chun 2008; Ali et al. 2012). Yet learners also require meaningful, everyday language interaction. Today’s university-level language learners face an increasing focus on independent learning using virtual platforms—at the expense of face-to-face learning hours (Bates 2015). The focus tends to be more on learning about a language than on learning to use the language as a vehicle for communication (Spada 1997; Berns et al. 2013b). As a result, learners are not given enough opportunities to interact and negotiate in the target language. Hence, conventional approaches alone often fail to meet basic learning needs.

The *VocabTrainerA1* app—designed specifically for this study—aims to address this reality by going beyond conventional approaches to provide students with a hybrid, game-based learning tool, combining individual and collaborative game tasks. Like the majority of available apps, *VocabTrainerA1* provides individual learners with valuable language input (Burston 2013, 2015) and the advantages of mobile learning. What differentiates the app—hence students’ experiences and learning outcomes—is the synergy created by combining individual learning tasks with an engaging collaborative role-play, in which learners are challenged to negotiate in the target language and use their language skills for real-world communication. This combination of individual and collaborative learning tasks—in a hybrid, level-based architecture—motivates and meets students’ needs more than conventional approaches, and has a positive impact on learning outcomes.

The qualitative data from the Technology Acceptance Model survey (TAM) and focus-group interviews confirm Hypothesis 1: *Students will be motivated by the use of a hybrid game*-*based app for learning purposes and will perceive a high degree of usefulness and added value*. The quantitative data from the conventional writing tasks, pre-test and post-test confirm Hypothesis 2: *Using a hybrid game*-*based app will have a positive impact on learning outcomes*.

In light of these findings, there is evidence that hybrid game-based apps like *VocabTrainerA1*—which seamlessly combine individual and collaborative learning tasks— motivate learners, stimulate perceived usefulness and added value, and better meet the language learning needs of today’s language learners. In terms of acceptance, outcomes and sustainability, the data suggests that, for today’s digital natives (Prensky 2001; Bates 2015), hybrid game-based apps significantly improve proficiency—hence are, indeed, effective tools for enhanced language learning.

With a view to harness the potential of the *VocabTrainerA1* app future work aims to further develop its content by increasing and diversifying the type of exercises and games included -hence providing learners with a wider range of opportunities to interact in the target language. This could be done by using similar games to those designed in order to improve other skills such as introductory computer programming competencies, etc. (Vahldick et al. 2014). Additionally, in order to provide a more detailed analysis of the different factors which could influence learning outcomes, when using hybrid game-based apps, the authors aim to test the *VocabTrainerA1* app with a much larger and diversified sample size (Experimental Group and Control Group) as well as for a longer period of time.

Future work should equally explore ways to harness the potential of computer-assisted assessment (CAA)—a key issue when dealing with high learner-to-teacher ratios. To this end, the authors aim to develop a Domain Specific Language (DSL) which would allow teachers to easily analyse in-app chat interaction logs and draw stronger conclusions regarding the relationship between student game behaviour and learning outcomes (Balderas et al. 2015). More specifically, CAA analysis of learner interaction would be an essential first step towards assessing learning outcomes, not only through pre-test and post-test evaluation but through the observation of the learning process itself—a growing demand on the part of educators and researchers alike (Bellotti et al. 2013; Burston 2015).

Another area of concern is the acceptance and sustainability of hybrid game-based apps like *VocabTrainerA1* in the short to mid-term (Berns et al. 2015). The authors propose further work on app design following the 3-phase iterative development process (experimentation-evaluation-enhancement) put forth by Nelson and Oliver (1999) and Kennedy and Levy (2009); specifically in terms of improving accessibility across different platforms and making the in-app chat more user-friendly.

## Appendix 1

See Table 7.

**Table 7:** TAM survey results (all averages calculated on a Likert scale of 1–5)

Questions	Average	Std dev.
1. Mobile App content and design		
1.1 The app’s content is interesting	4.47	0.70
1.2 The app’s content meets my learning needs	4.32	0.82
1.3 The ongoing feedback provided by the app is very useful	4.51	0.80
1.4 In general, I am satisfied with the content, design and quality of the app	4.48	0.63
2. Perceived Usefulness		
2.1 The app was useful for learning new vocabulary	4.83	0.48
2.2 The app helped me to improve my writing skills	4.51	0.67
2.3 The app helped me to improve my reading skills	4.27	0.81
2.4 The app was useful for learning to communicate more fluently in the target language	4.26	0.48
2.5 The app helped me to improve my overall language skills	4.55	0.68
3. Perceived Interaction		
3.1 The in-app text chat was effective for interacting with my game partners	4.44	0.86
3.2 The app provides valuable opportunities to communicate and negotiate in the target language	4.65	0.55
4. User Interface Design (UID)		
4.1 App layout/design make it very easy to use	4.37	0.66
4.2 The interface makes it easy to read and understand the information	4.34	0.65
4.3 The interface makes it easy to write	3.79	0.95
4.4 In general, I am satisfied with the design of the interface.	4.32	0.69
5. Perceived Ease of Use (PEU)		
5.1 The app was easy to use to play the mini-games (Levels 1–3)	4.20	0.76
5.2 The app was easy to use to play the role-play game (Level 4)	4.02	0.84
5.3 Overall, the app was easy to use	4.41	0.76
6. Intention to Use Apps for Learning Purposes		
6.1 I would use this kind of app for more learning activities within the classroom	4.38	0.80
6.2 I would use this kind of app for more learning activities outside the classroom	4.58	0.62
6.3 I intend to use this kind of app more often to improve my language proficiency in German/other languages	4.51	0.60

## Appendix 2

See Table 8.

**Table 8:** Focus group interview questions

Question 1:	How was your experience with the app?
Question 2:	What did you like most about the app?
Question 3:	What was the app’s main challenge with regard to your language learning?
Question 4:	What does the app mean to your language learning?
Question 5:	Did the app engage you to study more beyond the classroom?
Question 6:	How did the app influence your opinion on using apps for your autonomous language learning?
Question 7:	What do you suggest to make the app more efficient for your language learning?

## Abbreviations

App: applications
CAA: computer-assisted assessment
CEFR: common European framework of references for languages
DSL: domain specific language
ECTS: European Credit Transfer and accumulation System
SPSS: statistical package for the social sciences
TAM: technology acceptance model
VLE: virtual learning environments

## Footnotes

1 In the interest of the reader students in-app text chat conversation was translated into English.

2 Software forge for *VocabTrainerA1*: https://bitbucket.org/matrunks/deutschuca.

3 In the interest of anonymity, the names of all participants have been omitted. All answers were translated into English.
